# Evaluation of the Antinociceptive Effect of Sesamin: Role of 5HT_1A_ Serotonergic Receptors

**DOI:** 10.3390/pharmaceutics17030330

**Published:** 2025-03-03

**Authors:** Roberto Camacho-Cruz, David Francisco Alcalá-Hernández, Juan Carlos Huerta-Cruz, Jesús Arrieta-Valencia, María Elena Sánchez-Mendoza, Francisco Javier Flores-Murrieta, Andrés Navarrete, Juan Gerardo Reyes-García, Héctor Isaac Rocha-González

**Affiliations:** 1 Escuela Superior de Medicina, Instituto Politécnico Nacional, Mexico City 11340, Mexico; rcamchoc1700@alumno.ipn.mx (R.C.-C.); jarrietav@ipn.mx (J.A.-V.); msanchezme@ipn.mx (M.E.S.-M.); fjfloes@ipn.mx (F.J.F.-M.); jgreyes@ipn.mx (J.G.R.-G.); 2 División de Ciencias Biológicas y de la Salud, Universidad Autónoma Metropolitana Unidad Iztapalapa, Mexico City 09340, Mexico; 3 Instituto Nacional de Enfermedades Respiratorias, Ismael Cosío Villegas, Mexico City 14080, Mexico; juanc.huerta@iner.gob.mx; 4 Departamento de Farmacia, Facultad de Química, Universidad Nacional Autónoma de México, Mexico City 04510, Mexico; anavarrt@unam.mx

**Keywords:** diclofenac, gabapentin, inflammatory pain, neuropathic pain, serotonergic receptors, sesamin

## Abstract

**Background/Objectives:** Sesame (*Sesamum indicum* L.) is used in folk medicine to treat painful disorders. Sesamin is the main lignan found in this plant; however, its antinociceptive potential has scarcely been studied. The aim was to investigate the antinociceptive effect of sesamin on inflammatory and neuropathic pain models, as well as the possible mechanism of action through which sesamin mediates its own antinociceptive effect. **Methods:** Formalin and carrageenan animal models were used to assess inflammatory pain, whereas an L5/L6-spinal-nerve-ligated rat model was employed to evaluate neuropathic pain. **Results:** Oral sesamin significantly reduced carrageenan-induced hyperalgesia and inflammation, formalin-induced nociception, and L5/L6-spinal-nerve-ligation-induced allodynia. Sesamin was more effective than diclofenac in the inflammatory pain models, but it was less effective than pregabalin in the neuropathic pain model. The antinociceptive effect of sesamin, in the formalin test, was prevented by the intraperitoneal administration of methiothepin (5-HT_1/5_ antagonist), but not by naltrexone (an opioid antagonist) or L-NAME (an NOS inhibitor). In addition, WAY-100635 (5-HT_1A_ antagonist), but not SB-224289 (5-HT_1B_ antagonist), BRL-15542 (5-HT_1D_ antagonist), and SB-699551 (5-HT_5A_ antagonist), impeded sesamin-induced antinociception. **Conclusions:** This study’s results support the use of sesamin to treat inflammatory pain disorders and suggest that 5-HT_1A_ receptors influence the antinociceptive effect of this drug.

## 1. Introduction

Sesame (*Sesamum indicum* L.) is a warm-season annual plant of the *Pedaliaceae* family. It grows up to 2 m in height and produces bell-shaped pendulous flowers with a soft color ranging from white to pink and purple, and its fruits are rectangular capsules of 2–3 cm in length and 6–12 mm in diameter, which open with a pop when the seed matures [1,2].

In folk medicine from around the world, sesame seed oil is widely useful to treat wounds, burns, inflammation and several pain disorders, such as dysmenorrhea, abdominal pain, earache, and migraine, among others [3,4]. Around 160 phytochemical molecules have been isolated from sesame seeds, including lignans, polyphenols, phytosterols, anthraquinones, triterpenes, and other compounds, which have been linked to the health advantages of the seed. Lignans are regarded as the primary active compounds present in sesame seeds, and approximately half of the lignans present in those seeds are sesamin [4].

Several studies have pointed out that sesamin may be an effective therapeutic agent in preventing tumor development and the progression of various types of cancer due to its anti-proliferative, pro-apoptotic, anti-metastatic, and pro-autophagocytic properties [5]. In addition, it has been suggested that sesamin may be a good candidate for treating cardiovascular diseases, diabetes, hypertension, atherosclerosis, acute kidney injury, and fatty liver disease since it has antioxidant, anti-inflammatory, anti-hypertensive, hypoglycemic, lipolytic, hypocholesterolemic, anti-atherogenic, and anti-thrombotic activities [6,7,8,9,10]; however, the potential of sesamin in pain treatment has scarcely been studied.

The antinociceptive effect of sesamin has been evaluated in ethanolic and ethyl acetate extracts of *Zanthoxylum armatum*. In these studies, the extracts were able to significantly diminish acetic acid-induced visceral pain and formalin-induced inflammatory pain in mice, but the extracts contained a mixture of between 6 and 9 lignans, in which sesamin was not the main lignan [11,12]. The antinociceptive effect of sesamin has been evaluated in models of phasic pain such as hot-plate and tail immersion, as well as in acetic acid-induced visceral pain, but this effect in inflammatory pain remains controversial because studies have shown conflicting results. Indeed, sesamin was able to reduce the nociceptive behavior induced by formalin in [13], but not that of complete Freund adjuvant-induced inflammatory pain in [14]. Furthermore, the potential of sesamin as an antinociceptive agent in the treatment of neuropathic pain is unknown, and although the antinociceptive effect of sesamin has been suggested in some pain models, the mechanism of action through which sesamin reduces nociceptive behaviors has not been investigated. To this end, some studies point out that sesamin modulates the serotonin level in the central nervous system to reduce anxiety in several animal models [15,16]. Serotonin is the major neurotransmitter involved in the descending modulation of spinal nociceptive neurotransmission, and its effect is mainly antinociceptive in the central nervous system, although this depends on the specific serotonin receptor that is activated. The serotonin receptor family consists of 14 members divided into 7 families, where 5-HT_1_ and 5-HT_5_ receptors are considered inhibitory receptors due to the fact that they are negatively coupled to adenylyl cyclase and hyperpolarize the neurons [17,18], thus being a potential target through which sesamin induces its antinociceptive effect.

Consequently, the current study aimed to evaluate the antinociceptive effect of sesamin in formalin- and carrageenan-induced inflammatory pain, as well as its effect in L5/L6-spinal-nerve-ligation-induced neuropathic pain in rats. In addition, the mechanism of action underlaying the antinociceptive effect of sesamin is investigated in the formalin test.

## 2. Materials and Methods

### 2.1. Animals

All the behavioral experiments were performed on adult female Wistar rats (180–220 g). Female rats were used based on the fact that previous experiments in our test conditions have not shown significant differences between both sexes [19,20]. Animals were obtained from the institutional vivarium at the National Institute of Respiratory Diseases, Ismael Cosio Villegas (INER). Rats were housed in controlled conditions with a 12 h light/dark cycle at room temperature (23–25 °C) and with free access to food and water. Rats were fasted overnight (12 h) with free access to water before experiments. Experiments were designed to reduce pain and suffering in animals. In addition, the number of animals utilized was the minimum necessary to identify a reliable statistical difference. Animals were randomized into treatment groups a priori using the RandBetween function in Excel. On the experimental day, rats were put in observation acrylic chambers for 30 min to allow them to acclimate to their surroundings and to reduce their stress. Rats were euthanized in a carbon dioxide chamber at the end of the experimental paradigm. Only the observer was unaware of the treatment given to each animal during the evaluation.

### 2.2. Drugs

Formaldehyde and carrageenan were purchased from Sigma Aldrich (St. Louis, MO, USA). Methiothepin (1-[10,11-dihydro-8-(methylthio)dibenzo[b,f]thiepin-10-yl]-4-methylpiperazine mesylate salt), L-NAME (Nω-nitro-L-arginine methyl ester hydrochloride), naltrexone ((5α)-17-(cyclopropylmethyl)-4,5-epoxy-3,14-dihydromorphinan-6-one hydrochloride), WAY100635 (N-[2-[4-(2-methoxyphenyl)-1-piperazinyl]ethyl]-N-2 pyridinylcyclohexanecarboxamide maleatesalt), SB224289 (1′-methyl-5-[[2′-methyl-4′(5-methyl-1,2,4-oxadiazol-3-yl)biphenyl-4-yl]carbonyl]-2,3,6,7- etrahydrospiro[furo[2,3-f]indole-3,4′-piperidine]hydrochloride), BRL15572 (4-(3-chlorophenyl)-α-(diphenylmethyl)-1-piperazineethanol hydrochloride), and SB699551 (N-[2- dimethylamino)ethyl]-N-[[4′-[[(2-phenylethyl)amino]methyl][1,1′-biphenyl]-4yl]-methyl] and clopentanepropanamide dihydrochloride) were acquired from Tocris Biosciences (Bristol, Avon, UK). Methiothepin (non-selective 5-HT antagonist) [21], L-NAME (non-selective nitric oxide synthase inhibitor) [22], naltrexone (non-selective opioid antagonist) [23], WAY100635 (5-HT_1A_ antagonist) [24], SB224289 (5-HT_1B_ antagonist) [25], BRL15572 (5-HT_1D_ antagonist) [26], and SB699551 (5-HT_5A_ antagonist) [27] were chosen based on their selectivity according to the literature. Formaldehyde and carrageenan were prepared in a 0.9% physiological saline solution. Due to sesamin and some antagonists being water-insoluble compounds, we decided to use a mixture of physiological saline solution/dimethyl sulfoxide (90:10 v/v) as the vehicle (control group) to suspend them. The oral administration of this vehicle did not significantly modify the nociceptive responses with respect to the oral administration of a physiological saline solution vehicle. All drugs were freshly prepared before experiments. Doses of sesamin, antagonists, and the inhibitor were selected on the basis of previous studies and pilot experiments in our laboratory [13,14,28,29,30].

### 2.3. Plant Material

Sesame seeds (*Sesamum indicum*) were purchased from the Central de Abasto in México City, Mexico. Sesamin was extracted according to the previously described procedure with some modifications [31]. In brief, the seeds (4 kg) were subjected to grinding and extraction under reflux with n-hexane (3L) for 3 h three times. The evaporation of the solvent in vacuum yielded 250 g of oil sesame. Then, the oil sesame was fractionated using silica gel column chromatography. Elution was started with 100% hexane, and the polarity was increased using hexane/ethyl acetate mixtures in proportions of 9:1 and 8:2 in a gradient elution technique. A total of 60 fractions of 50 mL each were collected and screened via thin-layer chromatography using a reference sample of sesamin (Sigma Aldrich, St. Louis, MO, USA). The fractions containing sesamin (fractions 40–60, 8:2 hexane/ethyl acetate) were pooled and subjected to a second column of silica gel eluted with the same gradient elution technique mentioned above. From the second column, a total of 50 fractions of 50 mL each were obtained, where fractions 30 to 37 (8:2 hexane/ethyl acetate) yielded a white solid, which was purified via re-crystallization with hot ethanol until the fractions were akin to colorless needles (2.5 g, 0.063%). The crystals had a melting point of 122–123 °C, which corresponded to sesamin. To confirm the structure and molecular weight (354.11 g/mol) of sesamin, an electrospray ionization (ESI) analysis was performed using a Bruker micrOTOF-Q II mass spectrometer (Bruker Daltonics, Billerica, MA, USA). Using this technique, the sample was dissolved in ethanol and directly injected into the spectrometer under the following conditions: a capillary potential of −4.5 kV, a dry gas temperature at 200 °C, a dry gas flow of 4 L/min, and a mass range of 500 to 3000 *m*/*z*. The mass spectrometry data were processed using PolyTools 1.0 (Bruker Daltonics, Billerica, MA) [32].

### 2.4. Formalin Test

To confirm the antinociceptive effect of sesamin in inflammatory pain and to evaluate its mechanism of action, we performed the formalin test [33]. For this purpose, the rats were placed in 20 cm diameter and 30 cm high acrylic observation chambers with mirrors behind for 30 min to allow them to adapt to their environment. After this period, rats received a subcutaneous injection of 1% formalin (50 μL) in the region dorsal of the right hind paw using a 30 g needle. The nociceptive behavior was quantified as the number of flinches of the injected paw at intervals of 1 min every 5 min for 60 min [34]. The formalin-induced paw pain score was divided into phase 1 (0–10 min) and phase 2 (10–60 min). In this nociceptive model, 12 h fasted rats received an oral dose of vehicle (1 mL/kg), sesamin (0.32, 1, 3.2, and 10 mg/kg), or diclofenac (1, 3.2, 10, and 32 mg/kg) 60 min prior to formalin injection. To discern the mechanism of action of sesamin, rats received two doses in total, namely an oral dose of 3 mg/kg sesamin 60 min before 1% formalin injection and an intraperitoneal dose of naltrexone (1 mg/kg), L-NAME (0.1 mg/kg), methiothepin (1 mg/kg), WAY100635 (30 μg/kg), SB224289 (0.3 μg/kg), BRL15572 (30 μg/kg), or SB699551 (30 μg/kg) 30 min before formalin injection.

### 2.5. Thermal Hyperalgesia

To evaluate the antihyperalgesic effect of sesamin, 12 h fasted rats received a single oral dose of vehicle (1 mL/kg body weight) or increasing doses of sesamin (3.2, 5.6, 10, and 32 mg/kg) or diclofenac (3.2, 5.6, 10, and 32 mg/kg) just prior to the plantar 1% carrageenan injection. Thermal hyperalgesia was measured via the thermal nociception test device (Torrey Pines Instruments, San Diego, CA, USA), as previously described [35]. In this model, the rats were placed individually into acrylic boxes on the glass surface of the device. Once the rats were acclimated for 30 min, a thermal nociceptive stimulus was manually placed under the right hind paw before and every half hour until 6 h after carrageenan injection (50 μL, i.pl.). In each measurement, a timer was automatically started when the thermal stimulus was placed under the paw, and it stopped when the rat removed the paw. Thus, the nociceptive time latency was defined as the time required for paw withdrawal. A time cut-off of 20 s was fixed to elude tissue damage.

### 2.6. Carrageenan-Induced Paw Edema

The anti-inflammatory effect of sesamin was evaluated with the aid of a plethysmometer (Plethysmometer 7150; Ugo Basile, Gemonio, Italy) according to the method previously described by Winder et al. [36]. The device consisted of two interconnected methacrylate tubes filled with 0.9% physiological saline solution: in the longer one (18 mm in diameter), the injected paw of the rat was introduced in each measurement, whereas the shorter one had a transducer that measured the volume displaced in the long tube. On the day of evaluation, 12 h fasted rats were orally administered with vehicle (1 mL/kg) or increasing doses of sesamin (3.2, 5.6, 10, and 32 mg/kg) or diclofenac (3.2, 5.6, 10, and 32 mg/kg). Immediately afterwards, the rats received an intraplantar injection of 1% carrageenan (50 μL) in the right hind paw to induce the edema. Paw volume was measured before and after 1, 2, 3, 4, 5, and 6 h of carrageenan injection. To determine the differences in paw volume in μL (ΔVolume) with respect to the basal, the paw was submerged up to the tibiotarsal joint every time.

### 2.7. L5/L6-Spinal-Nerve-Ligation-Induced Tactile Allodynia

To test the antinociceptive effect of sesamin in a neuropathic pain model, we used the tactile allodynia model described by Chaplan et al., 1994 [37]. To perform this procedure, rats received an intraperitoneal injection of a mixture of ketamine/xylazine (45 and 12 mg/kg, respectively) to induce anesthesia. Then, rats were placed under a stereoscopic microscope, and the vertebral column was exposed in its lumbar area localizing the right L5 and L6 spinal nerves, which were ligated with a 6-0 silk suture distal to the dorsal root ganglion. Sham-operated rats were subjected to the same procedure; however, nerves were not ligated. Immediately, incision was sutured, and the rats were housed until recovery (14 days). Animals with motor deficiency were not included in the behavioral evaluation.

On the evaluation day, 12 h fasted rats were put into a transparent acrylic box on a metal mesh platform for 30 min to allow them to acclimate to their surroundings. Later on, rats were orally administered with vehicle (1 mL/kg), sesamin (3.2, 10, 32, and 100 mg/kg), or pregabalin (0.03, 1, 10, and 32 mg/kg). Tactile allodynia was evaluated by measuring the 50% paw withdrawal threshold in response to a set of calibrated von Frey filaments (Stoleting Co., Wood Dale, IL, USA) between 0.41 g (3.9 mN) and 15.1 g (147.1 mN), according to the previously described up–down threshold technique [37,38]. In this technique, an initial filament of 2 g was presented in the middle part of the right hind paw plantar surface, avoiding the paw pad, for 5 s. The positive response, observed as an abrupt withdrawal of the paw, was then followed by the application of a thinner filament, or a thicker filament, if the paw was not removed from the mechanical stimulus. After a response change was found, the rat was evaluated four more times to identify a pattern of six responses. The 50% paw withdrawal threshold was measured at 0, 0.5, 1, 2, 3, 4, 5, and 6 h and was calculated using the following formula:

$$50\%\;\mathrm{paw}\;\mathrm{withdrawal}\;\mathrm{threshold}\;(g)=\;\frac{10^{\left(Xf+k\delta \right)}}{10,000}$$

where *Xf* is the last value of the von Frey filament used in log units, *k* is the tabular value for the pattern of the six responses [37], and *δ* is the mean difference between stimuli. All the rats included in this study responded to a basal stimulus of less than 4 g.

### 2.8. Statistical Analysis

In all the behavioral studies, data were recorded as the mean ± S.E.M. of 6–8 rats per experimental group. For the formalin test, the time courses of the number of flinches were used to calculate the AUC of phase 1 and phase 2 of the test. From the AUC, the % antinociception for each phase was calculated using the following equation.

$$\%\mathrm{Antinociception}=\;\frac{{\mathrm{AUC}}_{\mathrm{Vehicle}\;\mathrm{flinches}}-\;{\mathrm{AUC}}_{\mathrm{Test}\;\mathrm{flinches}}}{{\mathrm{AUC}}_{\mathrm{Vehicle}\;\mathrm{flinches}}}\;\times \;100$$

In the carrageenan-induced hyperalgesia and inflammation experiments, time courses of paw withdrawal latency time (s) or volume change (ΔV in µL) were generated, respectively. From time courses, the area under the curve (AUC) was obtained via the trapezoidal rule. Then, the % anti-inflammatory effect was calculated via the following formula.

$$\%\mathrm{Anti}-\mathrm{inflammatory}\;\mathrm{effect}=\;\frac{{\mathrm{AUC}}_{\mathrm{Vehicle}\;\mathrm{volume}}-\;{\mathrm{AUC}}_{\mathrm{Test}\;\mathrm{volume}}}{{\mathrm{AUC}}_{\mathrm{Vehicle}\;\mathrm{volume}}}\;\times \;100$$

In contrast, the percentage of maximum possible effect (%MPE) for antihyperalgesic effect was determined via the next equation.

$$\%\mathrm{MPE}=\;\frac{{\mathrm{AUC}}_{\mathrm{Test}\;\mathrm{latency}}-\;{\mathrm{AUC}}_{\mathrm{Vehicle}\;\mathrm{latency}}}{{\mathrm{AUC}}_{\mathrm{Cut}-\mathrm{off}\;20s\;\mathrm{latency}}-\;{\mathrm{AUC}}_{\mathrm{Vehicle}\;\mathrm{latency}}}\;\times \;100$$

For the tactile allodynia model, the AUC was obtained via the trapezoidal rule from the time courses of the 50% paw withdrawal threshold. After that, the %MPE was calculated via the following formula.

$$\%\mathrm{MPE}=\;\frac{{\mathrm{AUC}}_{\mathrm{Test}\;\mathrm{tactile}\;\mathrm{threshold}}-\;{\mathrm{AUC}}_{\mathrm{Vehicle}\;\mathrm{tactile}\;\mathrm{threshold}}}{{\mathrm{AUC}}_{\mathrm{Sham}\;\mathrm{tactile}\;\mathrm{threshold}}-\;{\mathrm{AUC}}_{\mathrm{Vehicle}\;\mathrm{tactile}\;\mathrm{threshold}}}\;\times \;100$$

Later on, dose–response curves were constructed for each experimental model. The data distribution was evaluated using the Kolmogorov–Smirnov test, whereas the equality of variances between groups was evaluated using the Brown–Forsythe test and Bartlett’s test. Statistical differences between groups were determined via one-way analysis of variance (ANOVA), followed by Tukey’s test. For all cases, the statistical difference was considered significant when the *p* value was less than 0.05. Effective dose 30 (ED_30_) or effective dose 50 (ED_50_) ± error standard of the mean was calculated for each dose–response curve via Hill’s equation. Graphs were constructed using GraphPad Prism 9.5.

## 3. Results

### 3.1. Sesamin Reduces Formalin-Induced Nociception in Rats

**Figure 1:**
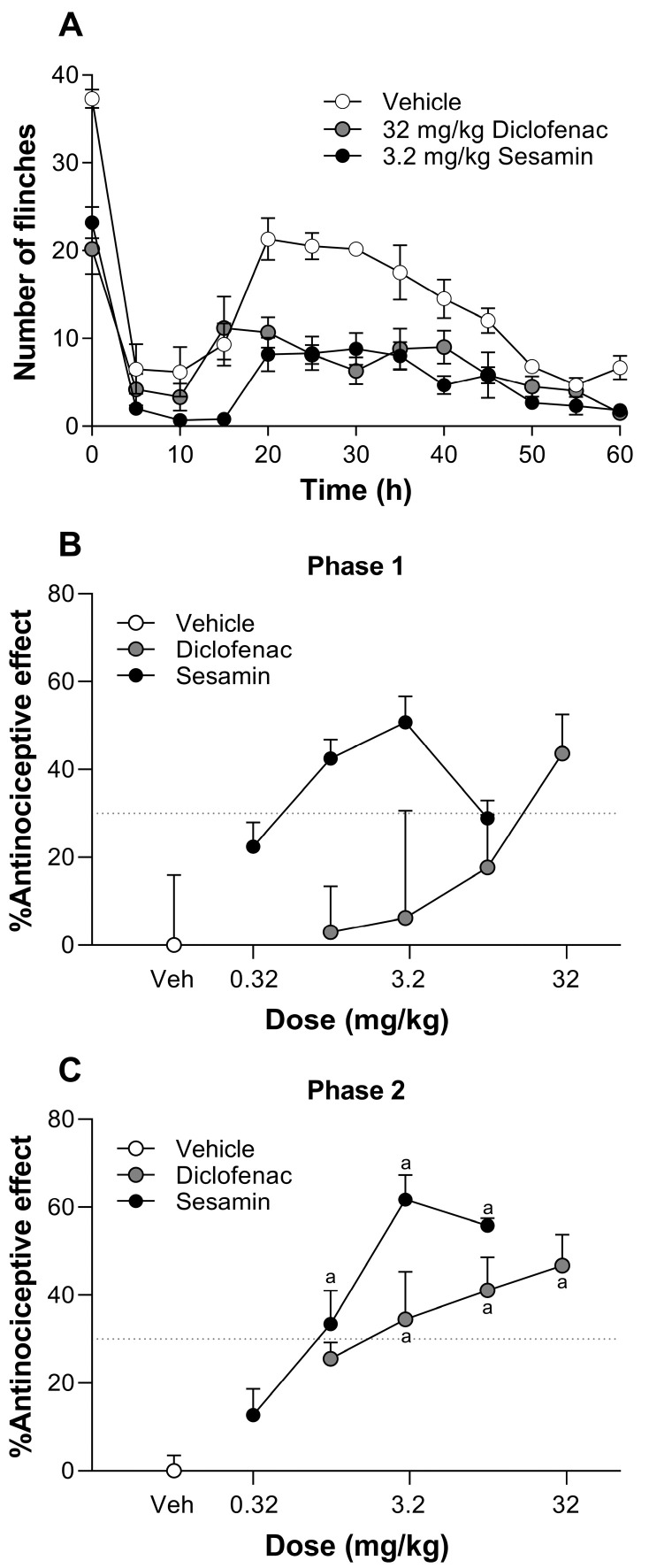
(**A**) The time course of the antinociceptive effect observed after the oral administration of sesamin (3.2 mg/kg) or diclofenac (32 mg/kg) in rats subjected to a subcutaneous injection of 1% formalin. (**B**,**C**) The dose–response curves of sesamin (0.32–10 mg/kg) and diclofenac (1–32 mg/kg) obtained during phase 1 and phase 2 of the formalin test in rats. The data are expressed as the mean ± S.E.M. from six rats per experimental group. (a) Significantly different from vehicle group (Veh) according to analysis of variance (ANOVA), followed by Tukey’s test.

The subcutaneous injection of 1% formalin into the dorsal surface of the right hind paw of a rat induced a spontaneous flinching behavior characterized by a typical biphasic time course. Phase 1 of the nociceptive behavior took place within 0–10 min after formalin injection, whereas phase 2 occurred with a duration of about 50 min (10–60 min), with a maximum value achieved at around 20 min. In this inflammatory pain model, the oral administration of both 3.2 mg/kg sesamin and 32 mg/kg diclofenac reduced formalin-induced flinching behavior with respect to the vehicle group along the time course (Figure 1A). In the dose–response curves, it is shown that sesamin significantly and dose-dependently reduced formalin-induced nociception between 0.32 and 3.2 mg/kg along phase 2, but only showed a decreasing trend during phase 1. Furthermore, sesamin attained its maximum effect at 3.2 mg/kg (61.8 ± 5.6%), and higher doses of sesamin did not produce a greater antinociceptive effect, showing an ED_30_ = 0.9 ± 1.8 mg/kg during phase 2. In a similar way, oral diclofenac significantly diminished the number of flinches induced by formalin in phase 2, but not in phase 1. Diclofenac presented an efficacy of 46.8 ± 7.0% at 32 mg/kg and an ED_30_ = 1.8 ± 2.8 mg/kg during phase 2. In the formalin test, sesamin was equieffective (*p* > 0.05) and equipotent (*p* > 0.5) to diclofenac (Figure 1B,C).

### 3.2. Oral Administration of Sesamin Reduces Carrageenan-Induced Hyperalgesia in Rats

**Figure 2:**
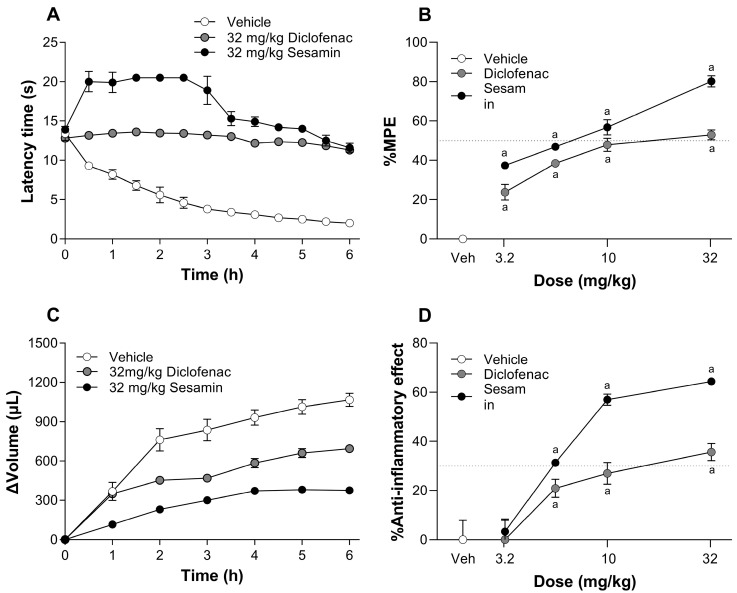
(**A**,**C**) The time course of the antihyperalgesic and anti-inflammatory effects observed after the oral administration of sesamin (32 mg/kg) or diclofenac (32 mg/kg) in rats subjected to an intraplantar injection of 1% carrageenan. (**B**,**D**) Dose–response curves obtained from the antihyperalgesic and anti-inflammatory effects induced by increasing oral doses of sesamin (3.2–32 mg/kg) or diclofenac (3.2–32 mg/kg) in rats subjected to an intraplanar injection of 1% carrageenan. Data are expressed as the mean ± S.E.M. from 6–8 rats per experimental group. (a) Significantly different from vehicle group (Veh) according to analysis of variance (ANOVA), followed by Tukey’s test.

The intraplantar injection of 1% carrageenan into the right hind paw gradually diminished the paw withdrawal latency time in the vehicle group (Figure 2A). On the contrary, the oral administration of sesamin (3.2–32 mg/kg) or diclofenac (3.2–32 mg/kg) dose-dependently prevented carrageenan-induced hyperalgesia (Figure 2B). In the time courses, 32 mg/kg of sesamin evoked its maximum effect at 0.5 h; this effect was maintained for about 2.5 h and then began to decline until reaching a basal value after 6 h. For its part, 32 mg/kg of diclofenac kept baseline latency times throughout the time course (Figure 2A). In this model, sesamin was more effective (*p* ˂ 0.001) than diclofenac at the tested doses because sesamin reached an efficacy of 80.2 ± 1.2%, whereas diclofenac achieved an efficacy of 52.9 ± 1.0 at the highest dose tested. Similarly, sesamin showed a lower potency (ED_50_ = 6.7 ± 1.9 mg/kg) than diclofenac (ED_50_ = 10.4 ± 1.5), but without statistical significance (*p* < 0.16). Both drugs were significantly different to the vehicle in the dose range of 3.2 to 32 mg/kg (Figure 2B).

### 3.3. Sesamin Orally Decreases Carrageenan-Induced Inflammation in Rats

In the vehicle group, the intraplantar administration of 1% carrageenan into the right hind paw increased the paw volume (Figure 2C). On the other hand, the oral administration of sesamin (3.2–32 mg/kg) or diclofenac (3.2–32 mg/kg) reduced carrageenan-induced inflammation in a dose-dependent manner (Figure 2D). In the time courses of volume that can be observed in the vehicle group, paw inflammation was progressively increased for at least 6 h. Meanwhile, oral sesamin (32 mg/kg) or diclofenac (32 mg/kg) reduced the gradual increase in paw volume induced by 1% carrageenan along the time course (Figure 2C). In the dose–response curves, it can be noticed that both sesamin and diclofenac achieved a significant anti-inflammatory effect from 5.6 mg/kg onwards. Sesamin was more potent (*p* < 0.02) and effective (*p* ˂ 0.001) than diclofenac in carrageenan-induced inflammation, since sesamin reached an efficacy of 64.3 ± 0.9% and had an ED_30_ = 5.5 ± 1.9 mg/kg, whereas diclofenac attained an efficacy of 35.6 ± 3.5% and an ED_30_ = 15.5 ± 3.3 mg/kg (Figure 2D).

### 3.4. Oral Administration of Sesamin Induces a Moderate Antiallodynic Effect in Rats Subjected to Spinal Nerve L5/L6 Ligation

**Figure 3:**
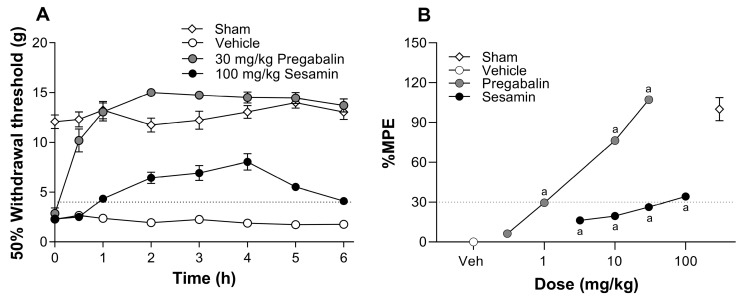
(**A**) The time course of the antiallodynic effect observed after the oral administration of sesamin (100 mg/kg) or pregabalin (30 mg/kg) in rats subjected to the ligation of spinal nerves L5/L6. (**B**) Dose–response curves of sesamin (3.2–100 mg/kg) or pregabalin (0.32–32 mg/kg) obtained in the L5-6-spinal-nerve-ligation-induced neuropathy model in rats. Data are expressed as the mean ± S.E.M. from six rats per experimental group. (a) Significantly different from vehicle group (Veh) via analysis of variance (ANOVA), followed by Tukey’s test.

Rats subjected to spinal nerve L5/L6 ligation showed reductions in the 50% withdrawal threshold to values less than 4 g, which was interpreted as tactile allodynia. In contrast, sham-operated rats maintained their 50% withdrawal threshold at values around 12 g. Allodynia was present in the rats from the first day following spinal surgery and persisted for at least 14 days, when the drugs were administered. The oral administration of 100 mg/kg sesamin slightly increased the 50% withdrawal threshold with a maximal antiallodynic effect at about 4h; then, the effect progressively decreased to basal values after 6 h. For its part, the oral administration of 30 mg/kg pregabalin gradually increased the 50% withdrawal threshold until achieving its maximum effect at around 2 h; then, the effect lasted for at least 6 h (Figure 3A). The oral administration of increasing doses of sesamin (3–100 mg/kg) resulted in a moderate dose–dependent antiallodynic effect. Conversely, oral pregabalin (0.3–30 mg/kg) dose-dependently increased its antiallodynic effect until reaching values close to 100% effect. In this model of neuropathic pain, pregabalin was clearly more effective (*p* ˂ 0.001) and potent (*p* ˂ 0.001) than sesamin, because pregabalin had an efficacy of 107 ± 1.6% and an ED_30_ = 9.8 ± 1.9 mg/kg, whereas sesamin presented an efficacy of 34.3 ± 2.0% and an ED_30_ = 56.5 ± 2.2 mg/kg. Both drugs were significantly different to the vehicle group in the tested dose range (Figure 3B).

### 3.5. Sesamin Reduced Formalin-Induced Flinching Behavior Through the Activation of 5-HT1A Receptors

**Figure 4:**
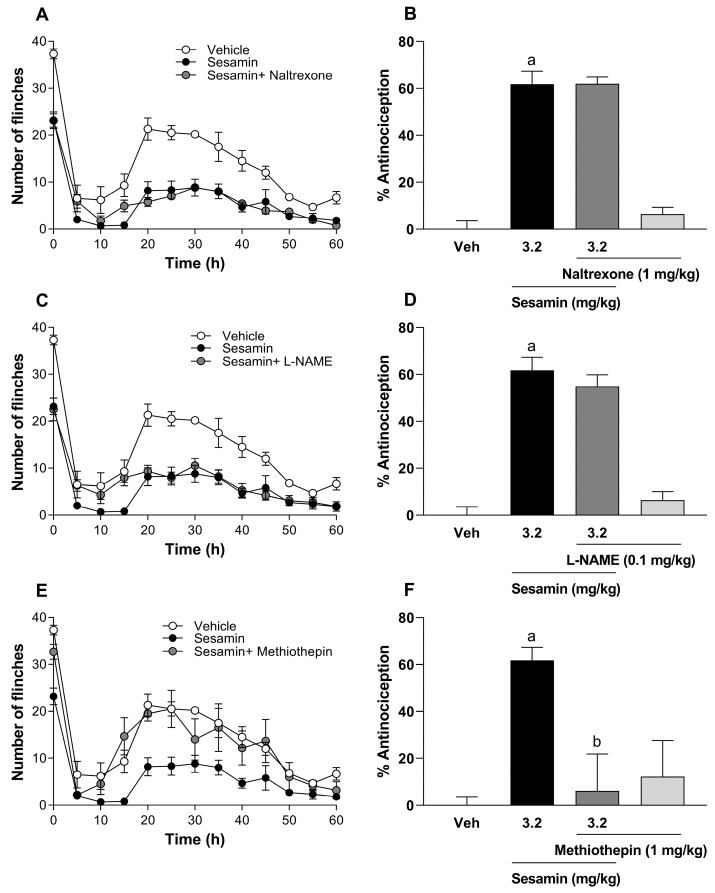
(**A**,**C**,**E**) The time courses of the antinociceptive effect observed after the oral administration of sesamin alone, sesamin and naltrexone, sesamin and L-NAME, or sesamin and methiothepin in the formalin test. (**B**,**D**,**F**) The effect of the intraperitoneal administration of naltrexone (1 mg/kg, −30 min), L-NAME (0.1 mg/kg, −30 min), or methiothepin (1 mg/kg, −30 min) on sesamin (3.2 mg/kg, −60 min)-induced antinociception in phase 2 of the formalin test. Bars are the mean ± S.E.M. from six rats per experimental group. (a) Significantly different from vehicle group (Veh); (b) significantly different from sesamin group via analysis of variance (ANOVA), followed by Tukey’s test.

The mechanism of action of sesamin was elucidated using the 1% formalin test. In this test, the intraperitoneal injection of 1 mg/kg naltrexone (non-selective antagonist opioid receptors; Figure 4A,B) or 0.1 mg/kg L-NAME (non-selective nitric oxide synthase inhibitor, Figure 4C,D) did not reduce the antinociceptive effect induced by the previous oral administration of sesamin (3.2 mg/kg) in phase 2. In marked contrast, the intraperitoneal injection of 1 mg/kg methiothepin (non-selective antagonist of 5-HT_1/5_ receptors, Figure 4E,F) blocked the antinociceptive effect exerted by sesamin in phase 2 of the formalin test. In this set of experiments, the doses of naltrexone (1 mg/kg), L-NAME (0.1 mg/kg), or methiothepin (1 mg/kg) did not modify the number of flinches induced by 1% formalin per se (Figure 4B,D,F).

**Figure 5:**
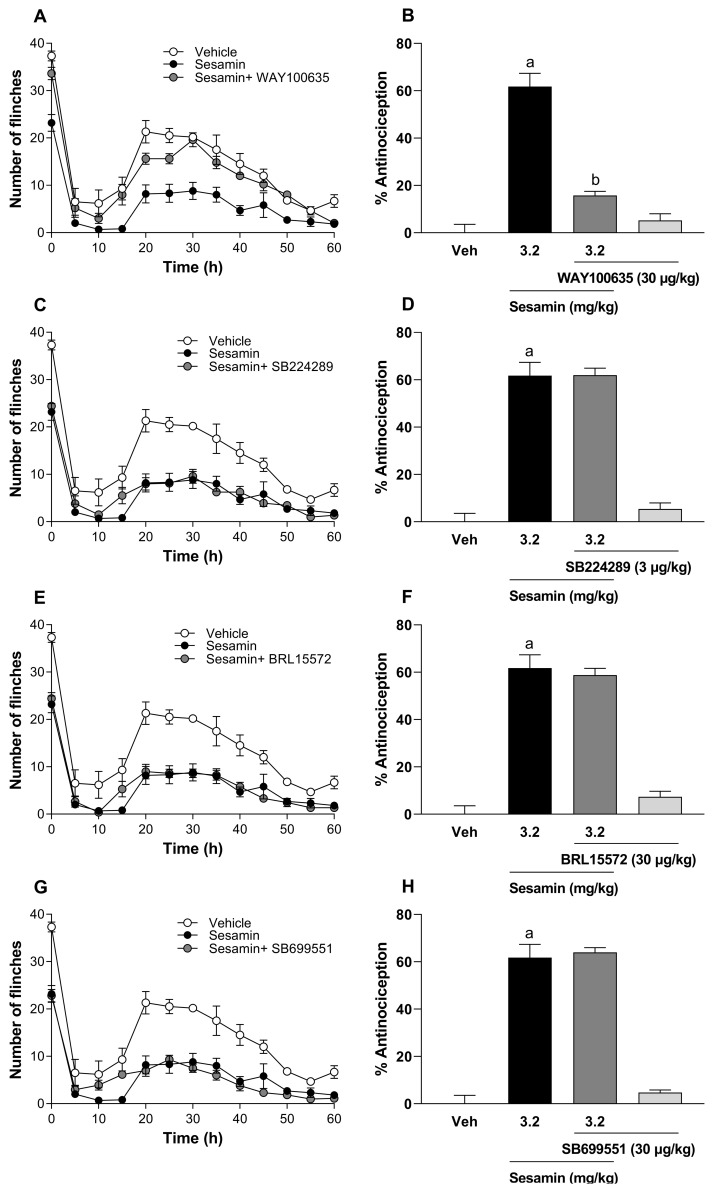
(**A**,**C**,**E**,**G**) The time courses of the antinociceptive effect observed after the oral administration of sesamin alone, sesamin and WAY100635, sesamin and SB224289, sesamin and BRL15572, or sesamin and SB699551 in the formalin test. (**B**,**D**,**F**,**H**) The effect of the intraperitoneal administration of WAY100635 (30 μg/kg, -30 min), SB224289 (3 μg/kg, -30 min), BRL15572 (30 μg/kg, -30 min), or SB699551 (30 μg/kg, -30 min) on sesamin (3.2 mg/kg, -60 min)-induced antinociception in phase 2 of the formalin test. Bars are the mean ± S.E.M. from six rats per experimental group. (a) Significantly different from the vehicle group (Veh); (b) significantly different from the sesamin group via analysis of variance (ANOVA), followed by Tukey’s test.

More selective antagonists of serotonin receptors were evaluated with sesamin because methiothepin reversed the antinociceptive effect of sesamin. In this way, only the intraperitoneal injection of 30 μg/kg WAY-100635 (5-HT_1A_ antagonist; Figure 5A,B) prevented sesamin-induced antinociception in phase 2 of the formalin test.

Unlike the intraperitoneal injection of 3 μg/kg SB224289 (5-HT_1B_ antagonist; Figure 5C,D), 30 μg/kg BRL15572 (5-HT_1D_ antagonist; Figure 5E,F) and 30 μg/kg SB699551 (5-HT_5A_ antagonist; Figure 5G,H) did not alter the decrease in the number of flinches induced by a previous oral administration of sesamin (Figure 5). The doses of WAY-100635 (30 μg/kg), SB-224289 (3 μg/kg), BRL-15572 (30 μg/kg), and SB-699551 (30 μg/kg) did not affect the nociceptive behavior induced by formalin in the rats per se, and such doses were selected on the basis of previous studies [28,29,30].

## 4. Discussion

This study aimed to characterize the antinociceptive potential of sesamin in various pain models in rats and to explore its mechanism of action. According to our results, increasing oral doses of sesamin showed a dose-dependent antinociceptive effect in the formalin-induced inflammatory pain model in rats. In line with our results, previous studies have demonstrated that some extracts from *Zanthoxylum* sp., containing sesamin, among other lignans, were able to reduce the time the mice spent licking the paw injected with formalin [11,12]. Furthermore, Monteiro et al. [13] pointed out that doses of sesame oil and sesamin between 100 and 200 mg/kg have the capacity to significantly reduce the paw-licking time induced by formalin in mice. In contrast with the doses used in mice, we observed that sesamin significantly reduced the formalin-induced number of flinches during phase 2 at doses between 10 and 100 times lower in rats. Such differences can be attributed to the species used, as it is well established that hepatic drug metabolism in mice is faster than that in rats [39,40]. Our results confirm the antinociceptive effect of sesamin in the formalin test and extend that effect to rats.

The antinociceptive effect of sesamin alone or contained in extracts has been suggested in models of phasic pain such as hot-plate or tail immersion, as well as in visceral pain models such as the acetic acid-induced writhing test, but it has only been demonstrated in the formalin test as an inflammatory pain model [11,12,13]. In fact, the chronic administration of sesamin did not decrease mechanical allodynia or thermal hyperalgesia in inflammatory pain induced by the hind paw injection of complete Freund adjuvant in mice [14]. In this regard, we decided to confirm the antinociceptive effect of sesamin in the carrageenan-induced inflammatory pain model in rats. Thus, the oral administration of increasing doses of sesamin dose-dependently reversed thermal hyperalgesia elicited by carrageenan. Taking these data together, we can confirm that sesamin is effective at reducing inflammatory pain induced by different stimuli. Regarding this point, we hypothesize that the lack of antinociceptive effect observed with complete Freund adjuvant, a model commonly used to mimic the nociceptive and inflammatory conditions of rheumatoid arthritis in an animal, is due to the dose used (80 mg/kg) in that study [14], rather than the model itself or ineffectiveness of sesamin, since it has been demonstrated that sesamin achieves a statistically significant antinociceptive effect at doses ≥ 100 mg/kg in mice [13]. In addition, a clinical study performed in 44 patients with rheumatoid arthritis demonstrated that 200 mg/day of sesamin supplementation for 6 weeks can diminish the number of tender joints and the severity of pain [41].

In our study, both in the formalin test and in the carrageenan model, sesamin was equipotent and more effective than diclofenac, the nonsteroidal anti-inflammatory drug (NSAID) most commonly prescribed worldwide to treat and manage pain associated with inflammatory conditions [42]. These results allow us to suggest that sesamin may be a therapeutic alternative for the treatment of inflammatory pain because it may have an advantage over diclofenac and other NSAIDs due to the scientific evidence that supports its beneficial effect on systems [7,9,43] that are traditionally affected by NSAIDs, such as cardiovascular, renal, hepatic, and gastrointestinal systems [44]. It is fair to say that the main limitation of this study is that it is a single-dose study; however, sesamin has been found in a repeat-dose study to treat rheumatoid arthritis in humans. In that study, 200 mg/day of sesamin for up to 6 weeks reduced the number of tender joints without any severe adverse events [41]. Thus, the evidence suggests that sesamin may be used as an adjuvant treatment to counterbalance the adverse effects of NSAIDs, which would allow the administration of these drugs for longer periods without sacrificing their analgesic efficacy.

Our results also indicate that the oral administration of sesamin produced an anti-inflammatory effect in the rats subjected to carrageenan injection. Concordantly, Monteiro et al. [13] previously reported similar results in this model. Although the mechanism of anti-inflammatory action of sesamin has not been evaluated directly in the paw injected with carrageenan, the evidence suggests that sesamin inhibits the infiltration of leucocytes and decreases the exudate volume in a carrageenan-induced lung inflammation model [13,45]; such effects seem to be associated with reductions in the expression of interleukin (IL)-1β, IL-8, tumor necrosis factor (TNF)-α, and macrophage-inflammatory protein 2 [45]. In another study, the daily oral administration of sesamin for 6 weeks improved the number of tender joints in patients with rheumatoid arthritis through reductions in serum levels of C reactive protein, TNF-α, and cyclooxygenase-2 [41].

On the other hand, to establish the antinociceptive potential of sesamin in a neuropathic pain model, we decided to measure the antiallodynic effect of sesamin in spinal-nerve-ligated (L5/L6) rats. In this model, sesamin produced a moderate reduction in allodynia, which was lower in potency and efficacy than that with pregabalin, a voltage-gated calcium channel blocker used to treat pain linked to spinal cord injury and other neuropathic pain disorders [46]. To the best of our knowledge, this is the first report which shows the antineuropathic effect of sesamin; although this effect was moderate, we think that the potential of sesamin should be evaluated in other neuropathic pain conditions that take advantage of its pharmacological properties. In this respect, painful diabetic neuropathy is caused by chronic high blood glucose, which leads to accumulating reactive oxygen species, inflammation, and cellular damage [47]. As a result, sesamin may offer a more comprehensive treatment of diabetic neuropathy because it has antioxidant [7], anti-inflammatory [10], hypoglycemic [6], and antineuropathic properties (this study).

To date, there have been no previous reports that analyze the mechanism of the antinociceptive effect of sesamin. Thus, the mechanism of action of sesamin was assessed in the formalin test because it was more effective in inflammatory pain than neuropathic pain. In a first set of experiments, we decided to administer L-NAME (nitric oxide synthase inhibitor), naltrexone (non-selective opioid receptor antagonist), and methiothepin (non-selective serotonin receptor antagonist) to evaluate nitrergic, opioidergic, and serotonergic systems, respectively, since they are common targets for natural products [27,48]. In the current study, the intraperitoneal injection of methiothepin was able to reverse the antinociception induced by sesamin in phase 2 of the formalin test. On the contrary, naltrexone or L-NAME did not do so. These results suggested that the serotonergic pathway, but not the opioidergic or nitrergic ones, is implicated in the antinociceptive effect of sesamin. In addition, our data are in line with anxiety studies, where it is shown that sesamin is capable of modulating the serotonin level in the brain [15,16].

To investigate the type of serotonin receptors involved in the antinociceptive effect of sesamin, we selected 5-HT_1A/B/D_ and 5-HT_5A_ receptors, because their transduction mechanism is negatively coupled to adenylyl cyclase and their activation opens and closes K^+^ and Ca^2+^ channels, respectively, hyperpolarizing neurons [17,18], which, in turn, are predominantly antinociceptive in pain animal models [17,18,30,49].

The data show that WAY100635 (5-HT_1A_ receptor antagonist) decreased the sesamin-induced antinociceptive effect in the formalin test. In marked contrast, SB224289 (5-HT_1B_ receptor antagonist), BRL15572 (5-HT_1D_ receptor antagonist), and SB699551 (5-HT_5A_ receptor antagonist) had no effect on antinociception induced by sesamin. The results strongly suggest that the antinociceptive effect of sesamin is mediated thought the activation of 5-HT_1A_ receptors, but not 5-HT_1B_, 5-HT_1D_, or 5-HT_5A_ receptors. Nonetheless, the participation of D4 receptors cannot be completely discarded because WAY100635 also binds to these receptors [50]. In line with our results, it is well known that the main source of serotonin in the central nervous system is localized in the nucleus raphe magnus, which sends descending projections to the spinal cord, where the 5-HT_1A_ receptor seems to be the predominant receptor for exerting the antinociceptive effect of serotonin [51,52,53]. In addition, the selective activation of 5-HT_1A_ receptors at the peripheral level leads to antinociception in the formalin test [30]. Because the expression of 5-HT_1A_ receptors has been observed on dorsal root ganglia [54] as well as in the spinal cord and brain structures as mesencephalic raphe nuclei and several amygdaloidal and brainstem nuclei [55], we speculate that the antinociceptive effect of sesamin may involve the peripheral and central activation of 5-HT_1A_ receptors.

## 5. Conclusions

In summary, our results confirm the antinociceptive effect of sesamin in inflammatory pain models in rats and evaluate, for the first time, its antiallodynic effect in a neuropathic pain test. The evidence suggests that sesamin is one of the main substances responsible for conferring anti-inflammatory and antinociceptive activities to the sesame plant, and this effect seems to be carried out through the activation of 5-HT_1A_ receptors. In addition, this study validates the traditional use of sesame oil in the treatment of inflammatory pain disorders such as dysmenorrhea, abdominal pain, and migraine. Finally, sesamin may be useful alone or as an adjuvant in the treatment of painful disorders in human beings.

## Data Availability

The original contributions presented in this study are included in this article; further inquiries can be directed to the corresponding author.
